# Class III *β*-Tubulin Overexpression Induces Chemoresistance to Eribulin in a Leiomyosarcoma Cell Line

**DOI:** 10.1155/2018/8987568

**Published:** 2018-06-21

**Authors:** Kenichiro Yahiro, Yoshihiro Matsumoto, Jun-ichi Fukushi, Ken-ichi Kawaguchi, Makoto Endo, Nokitaka Setsu, Keiichiro IIda, Suguru Fukushima, Makoto Nakagawa, Atsushi Kimura, Yoshinao Oda, Yasuharu Nakashima

**Affiliations:** ^1^Department of Orthopaedic Surgery, Graduate School of Medical Sciences, Kyushu University, 3-1-1 Maidashi, Higashi-ku, Fukuoka City 812-8582, Japan; ^2^Division of Orthopaedic Surgery, National Cancer Center Hospital, Tokyo, Japan; ^3^Department of Anatomic Pathology, Pathological Sciences, Graduate School of Medical Sciences, Kyushu University, Fukuoka, Japan

## Abstract

Eribulin is a new drug to treat soft tissue sarcoma (STS) that exerts antitumor activity by binding to microtubules. The prognosis of STS is poor, and eribulin is expected to improve the treatment outcome. We observed several cases that exhibited resistance to eribulin and developed an eribulin-resistant leiomyosarcoma cell line to investigate the mechanism of resistance. The IC50 of eribulin was 125 times higher in the resistant cell line than in the parental cell line, and eribulin did not induce G2/M arrest in resistant cells. The resistant cell line showed increased expression of MDR1 transcript, but protein levels and functional analysis results were similar to the parental cell line. We found that class III *β*-tubulin (TUBB3) was overexpressed in the resistant cell line, and siRNA knockdown of TUBB3 partially recovered sensitivity to eribulin. TUBB3 expression in clinical samples varied, suggesting that TUBB3 has the potential to be a biomarker for selection of anticancer drugs and may be a target for overcoming resistance to eribulin.

## 1. Introduction

The prognosis of high-grade malignant soft tissue sarcoma (STS) arising from a deep compartment is poor because of the high frequency of distant metastasis. To improve the prognosis of high-grade STS, systemic chemotherapy is necessary; however, there are few drugs for STS currently available [1]. Eribulin, a halichondrin B analog extracted from the marine sponge *Halichondria okadai* [2], is a new drug recently approved to treat STS [3]. Eribulin exerts antitumor activity by binding to the microtubules of tumor cells and inhibiting appropriate microtubule polymerization, which induces G2/M arrest of the cell cycle and leads to apoptosis [4].

In a nonrandomized, multicenter phase III trial [5], eribulin showed clinical efficacy for patients with relapsed malignant STS who had previously been treated with at least two regimens, including an anthracycline drug. The trial enrolled 452 patients with leiomyosarcoma (*n* = 297, 65.7%), liposarcoma (*n* = 153, 33.8%), and other sarcomas (*n* = 2, 0.4%). Eribulin significantly extended their overall survival (OAS) compared with dacarbazine (median OAS for the eribulin group was 13.5 months and 11.5 months for the dacarbazine group, *P* = 0.0169). Remarkably, eribulin showed superior efficacy in specific histological subtypes such as leiomyosarcoma and liposarcoma, and this result was confirmed by a subsequent clinical trial [6].

Leiomyosarcoma tumors consist of spindle-shaped cells with an intersecting fascicular growth pattern and are considered to stem from smooth muscle cells. As with other STSs, the prognosis of deep-seated leiomyosarcoma is poor, and eribulin would be a promising drug. However, we observed several cases of leiomyosarcoma that showed resistance to eribulin. In this study, we developed an eribulin-resistant leiomyosarcoma cell line to investigate the mechanism underlying resistance.

## 2. Material and Methods

### 2.1. Cell Lines and Culture Conditions

Human leiomyosarcoma cell line SK-LMS-1 was obtained from ATCC® (HTB-88™, Manassas, Virginia, USA) and maintained in Dulbecco's modified Eagle's medium (DMEM, Thermo Fisher Scientific, Waltham, Massachusetts, USA) supplemented with 10% fetal bovine serum (HyClone Laboratories, Logan, UT, USA), 100 units per ml penicillin, and 100 *μ*g per ml streptomycin at 37°C in an atmosphere of 5% CO_2_.

### 2.2. Establishment of Drug-Resistant SK-LMS-1 Cells

Eribulin mesylate (Halaven®) was purchased from Eisai (Tsukuba, Japan). Drug-resistant SK-LMS-1 cells were produced by selection in the presence of eribulin. Eribulin concentration was increased stepwise from 0.5 to 100 nM. Eribulin-resistant SK-LMS-1 cells were maintained in conditioned medium with 100 nM eribulin.

### 2.3. Chemosensitivity Assay and Doubling Time Measurement

Parental and drug-resistant SK-LMS-1 cells were seeded into a 96-well plate at a density of 2 × 10^3^ cells per well. After 24 hours of incubation, various concentrations of eribulin (0, 0.1, 0.5, 1, 5, 10, and 100 nM), vinblastine (0, 1, 5, 10, 50, 100, and 1000 nM), paclitaxel (0, 0.1, 0.5, 1, 5, 10, and 100 nM), and doxorubicin (0, 10, 100, 500, 1000, 5000, and 10,000 nM) were added to the media. After 48 hours of incubation with drugs, the number of viable cells in each well was measured using the CellTiter-Glo™ Luminescent Cell Viability Assay (Promega, Madison, WI, USA). Before the chemosensitivity assay, eribulin-resistant SK-LMS-1 cells were maintained without eribulin for 10 days. The chemosensitivity assay was carried out in triplicate.

Parental, resistant, and revertant SK-LMS-1 cells were seeded into a 96-well plate at a density of 2 × 10^3^ cells per well. After 1, 24, and 48 hours of incubation, the number of viable cells in each well was measured using the CellTiter-Glo Luminescent Cell Viability Assay.

### 2.4. Quantitative PCR

RNA was extracted using the RNeasy Mini Kit (Qiagen, Hilden, Germany) and reverse transcribed with PrimeScript™ RT Reagent Kit (Takara Bio, Kusatsu, Shiga, Japan). Real-time quantitative PCR was carried out with a LightCycler 1.5 (Perfect Real Time, Takara Bio, Kusatsu, Shiga, Japan), under conditions described previously [7]. The following primers were used: TUBB1 (forward: 5′-CCCCATACATACCTTGAGGCGA-3′ and reverse: 5′-GCCAAAAGGACCTGAGCGAA-3′), TUBB3 (forward: 5′-ATGCGGGAGATCGTGCACAT-3′ and reverse: 5′-CCCCTGAGCGGACCATGT-3′), TUBB4 (forward: 5′-GCTGTTTGTCTACTTCCTCCTGCT-3′ and reverse: 5′-CAGTTGTTCCCAGCACCACTCT-3′), and TUBB5 (forward: 5′-CGGGGAGGAAGCTTTTGAGGAT-3′ and reverse: 5′-CTGGGTAGAACCCGCAATTCTCT-3′). Data were standardized using GAPDH as a housekeeping gene. The assay was performed in triplicate, and three independent experiments were performed.

### 2.5. Western Blot Analysis

Normal and eribulin-resistant SK-LMS-1 cells were washed twice with ice-cold PBS, scraped, and centrifuged in microcentrifuge tubes. Cells were lysed using CelLytic M (Sigma-Aldrich, St. Louis, MO, USA) with a protease inhibitor (cOmplete™ Mini; Sigma-Aldrich, St. Louis, MO, USA) and phosphatase inhibitor (PhosSTOP; Roche Diagnostics, Mannheim, Germany) cocktail. Western blot analysis was performed as described previously [8], with the following primary antibodies: TUBB3 (1 : 100, clone TUJ1, BioLegend, San Diego, CA, USA), Pgp (1 : 100, clone C219, Enzo Life Sciences, Farmingdale, NY, USA), *β*-actin (1 : 1000, MAB1501, Merck Millipore, Burlington, Massachusetts, USA), and GAPDH (1 : 1000, D16H11, Cell Signaling Technology, Danvers, Mass). Relative intensity was calculated using the ratio of signal intensity of each target proteins and internal controls by using CS Analyzer 3.0 (ATTO, Amherst, NY, USA).

### 2.6. Cell Cycle Analysis by Flow Cytometry

Cells were harvested with or without 10 or 50 nM eribulin, incubated at 37°C for 12 hours, washed with PBS, and fixed with 70% ethanol at 4°C for 30 minutes. Cells were then centrifuged and washed with PBS, and 0.5 ml propidium iodide solution (PI/RNase, Cosmo Bio Company, Tokyo, Japan) was added. Cells were incubated at room temperature for 15 minutes before analysis. Alterations in cell distribution were analyzed using BD Accuri™ C6 flow cytometer (BD Biosciences, San Jose, CA, USA). For each sample, 10,000 events were recorded.

### 2.7. TUBB3 siRNA Experiments

Transfection of siRNA was carried out according to the manufacturer's protocol.

SK-LMS-1 cell lines were seeded into 6-well plates at a density of 1 × 10^5^ cells per well and incubated at 37°C overnight without antibiotics. After incubation, cells were transfected with TUBB3 siRNA (s20296, Thermo Fisher Scientific, Waltham, Massachusetts, USA) or negative control siRNA (Silencer® Select Negative Control No. 1 siRNA, Thermo Fisher Scientific, Waltham, Massachusetts, USA) using Lipofectamine® 2000 (Thermo Fisher Scientific, Waltham, Massachusetts, USA). The introduction of siRNAs was confirmed by quantitative PCR and Western blotting. The chemosensitivity assay was performed 24 hours after transfection, as described above.

### 2.8. Functional Analysis of MDR1 as a Drug Transporter

A functional test of MDR1 was performed using the EFLUXX-ID® Green Multidrug Resistance Assay kit (Enzo Life Sciences, Farmingdale, NY, USA). Cells were incubated with FBS-free medium, washed with PBS, and resuspended in FBS-free medium. Single-cell suspensions were mixed with diluted MDR1 inhibitor at 37°C for 5 minutes, then all samples were incubated with diluted EFLUXX-ID Green Detection Reagent at 37°C for 30 minutes. After incubation, 5 *μ*l of propidium iodide was added to all samples and fluorescence was immediately measured using a BD Accuri C6 flow cytometer.

### 2.9. Ethics Guidelines

Our all work was conducted in accordance with the Declaration of Helsinki, and the experiment was conducted with the human subject's understanding and consent. The Institutional Review Board in Kyushu University in Fukuoka, Japan, has approved the experiments (approval number 26-224).

### 2.10. Patients and Immunohistochemistry

Immunohistochemical staining was performed as described previously [9]. We used the samples of soft tissue leiomyosarcoma registered in the files of the Department of Anatomic Pathology, Graduate School of Medical Sciences, Kyushu University, Fukuoka, Japan. 68 samples of soft tissue leiomyosarcoma from 68 patients were prepared for immunohistochemistry. These samples had been obtained from biopsy specimens or surgically resected tumors. Samples after chemotherapy were not included in immunohistochemistry. All samples were fixed in 10% neutral buffered formalin and embedded in paraffin. After being deparaffinized in xylene and dehydrated in a graded ethanol series, sections were pretreated with 1.0 nM EDTA (Wako Pure Chemical Industries, Osaka, Japan) in a microwave oven at 100°C for 15 minutes before being incubated with anti-TUBB3 monoclonal antibodies (1 : 100, clone TUJ1, BioLegend, San Diego, CA, USA) at 4°C overnight. Samples were then incubated with Dako EnVision Dual Link System-HRP (Agilent, Santa Clara, CA, USA), visualized using the diaminobenzidine substrate system (Wako Pure Chemical Industries, Osaka, Japan), and counterstained with diluted hematoxylin.

### 2.11. Statistical Analysis

Student's *t*-test was used for two-group comparisons. *P* < 0.05 was considered to be statistically significant. Data in graphs are given as means ± standard deviation (SD). The log-rank test was used for Kaplan-Meier survival estimate of clinical leiomyosarcoma samples. *P* < 0.05 was considered to be statistically significant. All statistical analyses were performed with the Statistical Analysis System (SAS) software package (JMP Pro 12, SAS Institute, Cary, NC, USA).

## 3. Results

### 3.1. Establishment of Eribulin-Resistant SK-LMS-1 Cell Line and Cell Cycle Analysis

**Figure 1:**
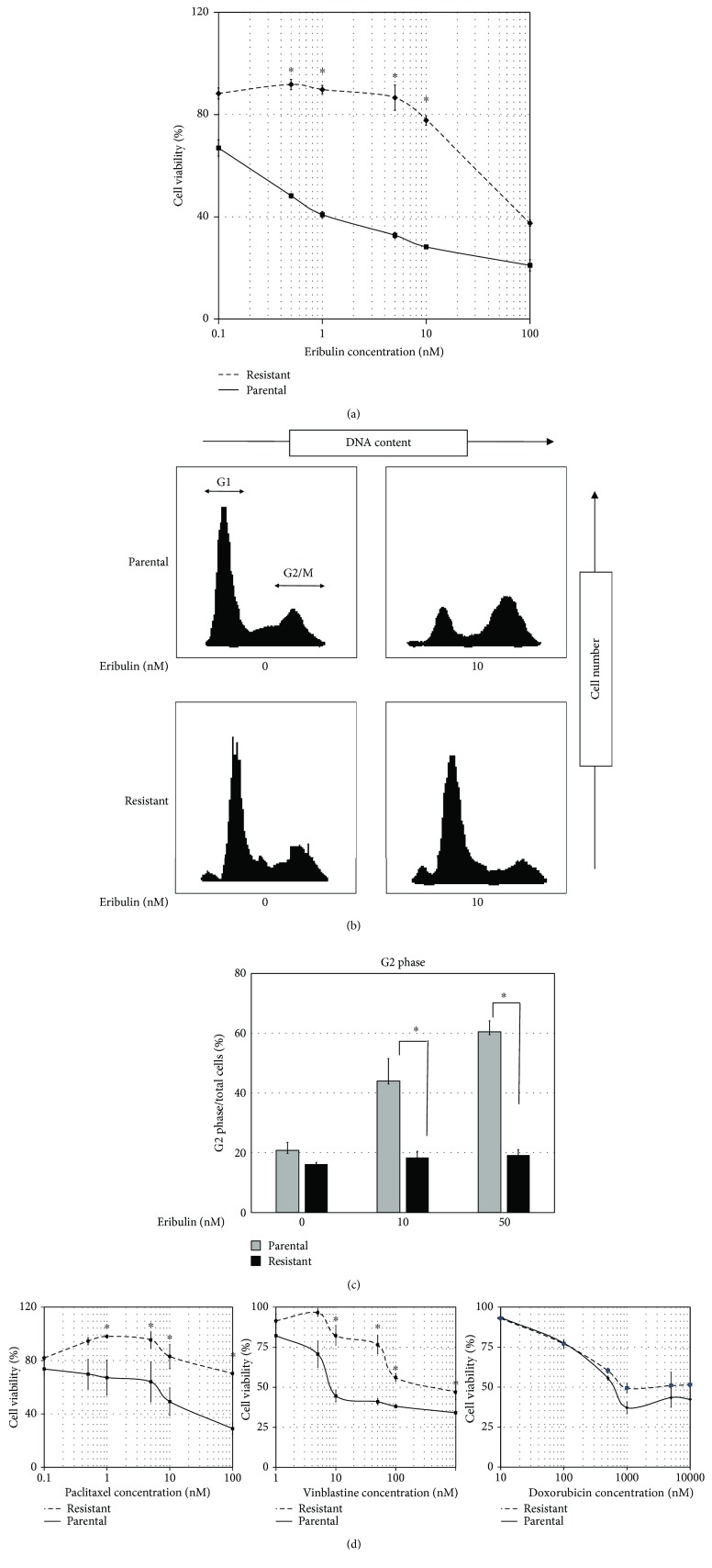
Drug sensitivity and cell cycle analysis of parental and eribulin-resistant cell lines. (a) The leiomyosarcoma cell line was incubated with various doses of eribulin for 48 hours. The solid line represents the parental cell line, and the dotted line represents the eribulin-resistant cell line. Viable cells were measured using CellTiter-Glo. Values represent mean ± SD. ^∗^ *P* < 0.05, versus parental cell line. (b, c) Cells were incubated with eribulin (0 nM, 10 nM, or 50 nM) for 12 hours and fixed with 70% ethanol. The DNA content of each phase was analyzed by flow cytometry after staining with PI, and the percentage of cells in G2 phase was calculated. Values represent mean ± SD. ^∗^ *P* < 0.05, versus parental cell line. (d) The leiomyosarcoma cell line was also incubated with various doses of paclitaxel (left panel), vinblastine (middle panel), and doxorubicin (right panel). The solid line represents the parental cell line, and the dotted line represents the eribulin-resistant cell line. Viable cells were measured using CellTiter-Glo. Values represent mean ± SD. ^∗^ *P* < 0.05, versus parental cell line.

SK-LMS-1 cells were subjected to stepwise increases in the concentration of eribulin up to a final concentration of 100 nM, and development of eribulin resistance was confirmed periodically using a chemosensitivity assay. After 2 months, a stable eribulin-resistant clone was established and we confirmed chemoresistance to eribulin (Figure 1(a)). We found the resistant cell line to be approximately 125 times more resistant to eribulin than the parental cell line (IC50 of 50 nM versus 0.42 nM for the parental line) (Figure 1(a)).

The proposed mechanism of eribulin's anticancer activity is inhibition of the growth of microtubules, thereby inducing G2/M arrest. We utilized flow cytometry to analyze the effect of eribulin on the cell cycle of the eribulin-resistant and parental SK-LMS-1 cell lines. We observed that the parental cell line consisted of 64.4 ± 3.2% G1 cells and 20.8 ± 2.7% G2 cells in the absence of eribulin and consisted of 23.0 ± 3.8% G1 cells and 60.5 ± 3.7% G2 cells in the presence of 10 nM eribulin, indicating that eribulin induced G2/M arrest. The resistant cell line consisted of 69.3 ± 1.4% G1 cells and 16.2 ± 0.6% G2 cells in the absence of eribulin and consisted of 64.6 ± 3.6% G1 cells and 19.2 ± 1.9% G2 cells in the presence of 10 nM eribulin. These results demonstrate that eribulin does not induce G2/M arrest in the resistant cell line (Figure 1(b)). We also incubated parental and resistant cell lines in the presence of 50 nM eribulin and analyzed the cell cycle of each cell line. The G2 cells of the parental cell line were gradually increased as the concentration of eribulin was higher, but those of the resistant cell line were not increased (Figure 1(c)).

### 3.2. Eribulin-Resistant SK-LMS-1 Cell Line Exhibits Cross-Resistance to Other Microtubule-Interacting Drugs but Not Anthracyclines

Because the previous study reported that TUBB3 overexpression induced drug resistance to some microtubule-interacting drugs such as taxanes or vinca alcaloids, we investigated whether our eribulin-resistant cell line exhibited cross-resistance to other microtubule-interacting drugs and anthracyclines which were used as standard treatment for sarcoma. According to previous studies, the eribulin-resistant cell line exhibited cross-resistance to paclitaxel and vinblastine but not anthracyclines (Figure 1(d)).

### 3.3. Expression and Functional Analysis of MDR1 in Eribulin-Resistant Cells

**Figure 2:**
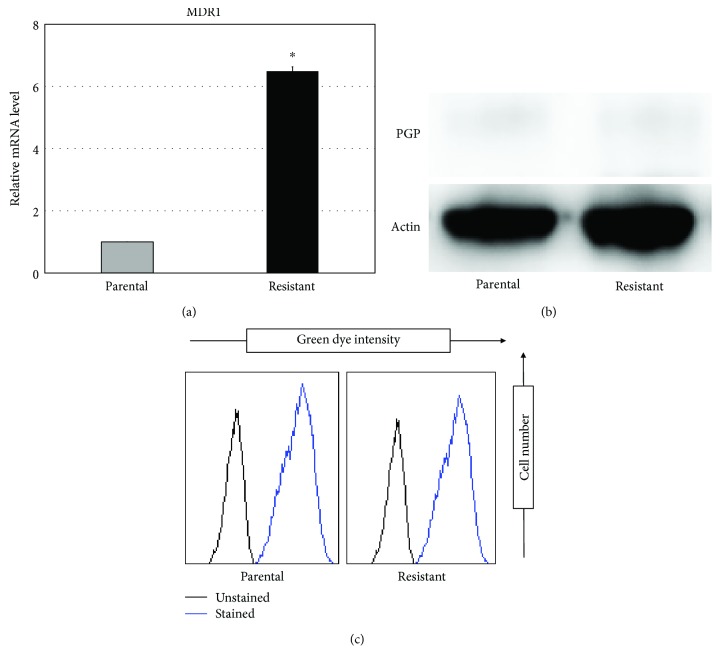
Expression and functional analysis of MDR1. (a, b) The expression of TUBB3 was investigated by real-time quantitative PCR and Western blotting in each cell line. Values represent mean ± SD. ^∗^ *P* < 0.05, versus parental cell line. (c) Functional activity of MDR1 was analyzed by EFLUXX-ID Green Multidrug Resistance Assay. The left black curve shows unstained cells as a negative control. The right blue curve shows cells stained with EFLUXX-ID Green.

One plausible scenario for eribulin resistance is increased expression of the ATP-dependent drug efflux pump P-glycoprotein 1 (Pgp) in resistant cells. We examined the expression of the multidrug resistance 1 (MDR1) gene, which encodes Pgp, in the parental and resistant cell lines using quantitative PCR and evaluated Pgp protein levels using Western blot analysis. In quantitative PCR assays, the eribulin-resistant cell line exhibited higher expression of MDR1 than did the parental cell line (Figure 2(a)). However, we did not detect a significant difference in protein levels between the parental and eribulin-resistant cell lines (Figure 2(b)). In addition, functional analysis of Pgp revealed that there was no significant difference in the efficiency of Pgp between the parental and eribulin-resistant cell lines (Figure 2(c)). Taken together, these results suggest that Pgp does not play a dominant role in the chemoresistance of our eribulin-resistant cell line.

### 3.4. Altered *β*-Tubulin Expression Profile May Induce Eribulin Resistance in SK-LMS-1 Cells

**Figure 3:**
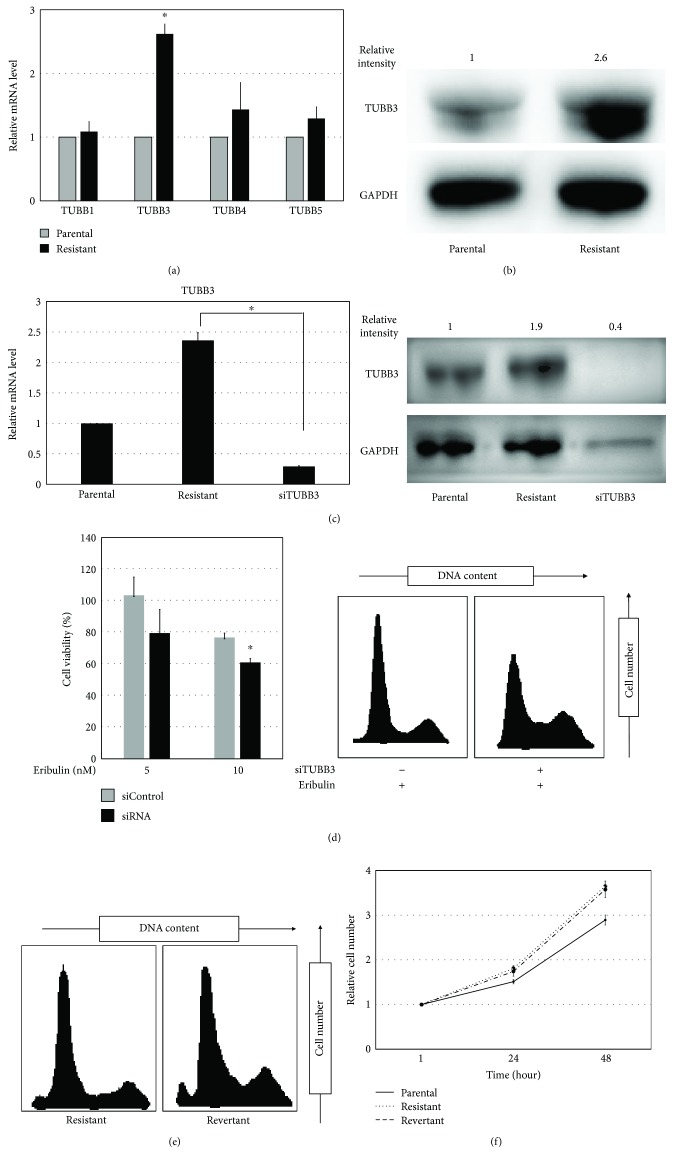
*β*-Tubulin profile and TUBB3 knockdown in parental and eribulin-resistant cell lines. (a) The *β*-tubulin profile (TUBB1, TUBB3, TUBB4, and TUBB5) was investigated by real-time quantitative PCR in each cell line. Values represent mean ± SD. ^∗^ *P* < 0.05, versus parental cell line. (b) Overexpression of TUBB3 was confirmed by Western blotting. GAPDH was used for internal normalization. (c) The expression of TUBB3 after transfection of siRNA was analyzed by real-time quantitative PCR and Western blotting. Values represent mean ± SD. ^∗^ *P* < 0.05. (d) The sensitivity to eribulin after transfection of siRNA was assessed by determination of cell viability and cell cycle analysis. Left panel: the cell viability assay was performed after transfection of siRNA and 48-hour incubation with eribulin. Right panel: cell cycle analysis was performed after transfection of siRNA and 12-hour incubation with eribulin. Values represent mean ± SD. ^∗^ *P* < 0.05, versus resistant cell line transfected with siControl. (e) Cell cycle analysis of the resistant cell line 4 weeks after incubation without eribulin. The left panel represents analysis of the resistant cell line. The right panel represents analysis of the revertant cell line which was incubated without eribulin for 4 weeks. There was no significant difference in each cell cycle, so chemoresistance to eribulin was maintained 4 weeks after incubation without eribulin. (f) The doubling time of three cell lines, parental, resistant, and revertant, was measured by using CellTiter-Glo. The solid line represents the parental cell line, the dotted line represents the resistant cell line, and the broken line represents revertant used in Figure 3(e). Values represent mean ± SD.

The main target of eribulin is microtubules, which are polymers composed of *α*- and *β*-tubulin dimers. Tubulin-binding drugs are known to bind *β*-tubulin, which has several isotypes. We speculated that alteration of the expression profile of *β*-tubulin isotypes might result in chemoresistance to eribulin. Quantitative PCR assays showed that the eribulin-resistant cell line had significantly higher *β*-tubulin 3 (TUBB3) expression than the parental cell line, whereas there were no significant differences in expression of TUBB1, TUBB4, or TUBB5 (Figure 3(a)). This result was confirmed by Western blotting (Figure 3(b)). To test whether suppression of TUBB3 expression might restore sensitivity to eribulin in our resistant cell line, we transfected resistant cells with siRNA targeting TUBB3. We confirmed knockdown of TUBB3 using quantitative PCR and Western blotting (Figure 3(c)). Importantly, knockdown of TUBB3 expression led to chemosensitization to eribulin in the resistant cell line (Figure 3(d)). These results indicate that TUBB3 overexpression may play a major role in chemoresistance to eribulin in the resistant cell line. To determine whether the chemoresistance was reversible, we incubated the resistant cell line without eribulin for 4 weeks, established the “revertant cell line,” and monitored whether the cells became sensitive to the drug. The revertant cell line maintained its chemoresistance to eribulin for 4 weeks after incubation without eribulin,demonstrating that the chemoresistance was irreversible (Figure 3(e)).

### 3.5. TUBB3 Expression of SK-LMS-1 Is Involved in Cell Proliferation Rate

We investigated the cell proliferation rate of parental, eribulin-resistant cells and revertants. The eribulin-resistant cells and revertants showed a slight trend toward extension of doubling time compared to parental cells; however, the difference was not significant (Figure 3(f)). Next, we inhibited the TUBB3 expression by siRNA in parental and resistant cell lines and investigated each cell proliferation rate. We found that the reduction of TUBB3 expression in the parental and resistant leiomyosarcoma cells resulted in a trend toward a decreasing cell proliferation rate, but the difference was not statistically significant (data not shown).

### 3.6. The Overexpression of TUBB3 Correlates with Poor Prognosis in Clinical Leiomyosarcoma Samples

**Figure 4:**
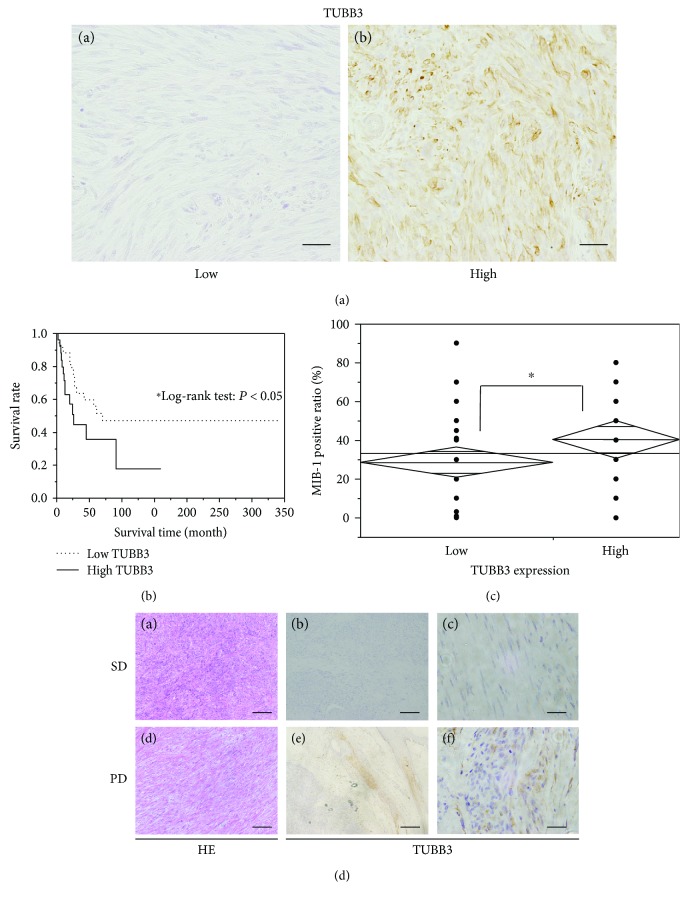
Expression of TUBB3 in clinical leiomyosarcoma samples. (a) Immunohistochemical staining of TUBB3 in 68 clinical samples from patients with leiomyosarcoma. (A, B) the representative samples of immunohistochemical staining of TUBB3: (A) low TUBB3 expression and (B) high TUBB3 expression. Scale bar represents 100 *μ*m in (A) and (B). (b) Overall survival analysis of the patients with leiomyosarcoma which express high or low TUBB3 by the Kaplan-Meier method. The solid line represents high TUBB3 group, and the dotted line represents low TUBB3 group. ^∗^ *P* < 0.05. (c) Analysis of TUBB3 expression and MIB-1 positive ratio (%). The high TUBB3 group exhibited a significantly higher MIB-1 positive ratio than did the low TUBB3 group. ^∗^ *P* < 0.05. (d) HE and immunohistochemical staining of TUBB3 in tumor biopsies from patients who were treated with eribulin. (A, B, C) Upper specimens represent the tissue samples from the patient who continued to have stable disease (SD) 12 weeks after initial treatment with eribulin. (D, E, F) Lower specimens represent the tissue samples from the patient who resulted in progressive disease (PD) after treatment with eribulin. (A, D) Staining with hematoxylin and eosin. (B, C, E, F) Immunohistochemical staining of TUBB3. Scale bar represents 100 *μ*m in (A) and (D), 200 *μ*m in (B) and (E), and 20 *μ*m in (C) and (F).

In order to examine the expression patterns of TUBB3 in clinical samples, we performed immunohistochemical staining of TUBB3. Remarkably, we found that the expression of TUBB3 was variable, with 27 cases out of 68 (39.7%) showing high expression of TUBB3, whereas the remaining 41 cases (60.3%) showed low expression of TUBB3 (Figure 4(a)). To investigate the relationship with TUBB3 expression and prognosis or cell growth (MIB-1 expression) in clinical samples, we investigated the overall survival time and MIB-1 expression of samples that we performed the immunohistochemical staining of TUBB3. We found that high TUBB3 expression significantly correlated with poorer overall survival (Figure 4(b), median overall survival time: low TUBB3 group; 70 months versus high TUBB3 group; 26 months, *P* < 0.05) and higher cell growth (Figure 4(c), MIB-1 positive ratio: low TUBB3 group; 28.6 ± 3.9% versus high TUBB3 group; 40.4 ± 4.8%, *P* < 0.05).

In addition, we analyzed tissue biopsy samples from two leiomyosarcoma patients treated with eribulin; one showed high levels of TUBB3 expression and the other showed low levels. We observed progressive disease in the patient with high expression of TUBB3, whereas the patient with low expression of TUBB3 continued to have stable disease 12 weeks after initial treatment with eribulin (Figure 4(d)). We also performed immunohistochemical staining of Pgp and investigated the Pgp expression in these clinical samples. As a previous report [10], we recognized slight Pgp expression in clinical samples, so we are not able to evaluate any relationship with Pgp expression and chemoresistance to eribulin (Supplementary Fig.  1).

## 4. Discussion

Drug resistance is one of the most serious problems in tumor therapy. Because there are few effective drugs for STS, therapeutic strategy for STS is strongly limited once drug resistance is established [11]. Understanding the mechanism of drug resistance is a critical step toward improving survival of STS patients. Previous studies have shown that a common mechanism of drug resistance in various tumors is overexpression of a drug efflux pump such as Pgp [12]. Eribulin is a potential substrate of Pgp, and some cell lines, including a breast cancer cell line, have acquired resistance to eribulin via overexpression of MDR1 and Pgp [13]. We observed an increased expression of MDR1 in our resistant cell line compared to the parental cell line; however, there were no significant differences in protein levels or functionality of Pgp. It is possible that Pgp plays a dominant role for chemoresistance to eribulin in breast cancer but not in leiomyosarcoma or there were errors in posttranscriptional processing of Pgp in the eribulin-resistant cell line, and the exact mechanism should be studied further.

Several classes of anticancer drugs inhibit cell division by acting upon microtubules, and mutations in tubulin or microtubule-associated proteins may induce drug resistance [14]. Taxanes bind to tubulin and stabilize microtubules by preventing depolymerization, resulting in cell cycle arrest and apoptosis [15, 16]. Vinca alkaloids bind to tubulin and inhibit polymerization of microtubules [17]. Eribulin binds to a unique site on tubulin and irreversibly inhibits microtubule growth [1]. As a result, cells cannot form mitotic spindles and arrest at the G2/M transition before eventually undergoing apoptosis. We found that administration of eribulin to the parental cell line induced G2/M arrest and apoptosis, but the resistant cell line did not undergo cell cycle arrest and continued to proliferate after administration of eribulin.

Alteration of the *β*-tubulin profile, particularly overexpression of the TUBB3 isotype, has been linked to drug resistance [18]. TUBB3 is mainly expressed in neurons and the testes [19], but there has been a report that some cancers overexpress TUBB3 and it may be involved in tumor aggressiveness and prognosis [20]. Importantly, Wilson et al. showed that eribulin had an increased binding affinity for microtubules in the absence of TUBB3 [21]. In line with this, we showed that high TUBB3 expression induced resistance to eribulin in a leiomyosarcoma cell line and we hypothesize that the resistance to eribulin stems from TUBB3 overexpression. Our resistant cell line which expressed high TUBB3 exhibited cross-resistance to other microtubule-interacting drugs, such as vinca alkaloids or taxanes. It is consistent with previous study.

The possible association of TUBB3 overexpression with cancer aggressiveness or metastasis has been reported for several cancers, including non-small-cell lung cancer [22], breast cancer [20], urinary bladder cancer [23], and esophageal adenocarcinoma [24]. However, the biological roles of tubulin, as well as expression profiles of tubulin isotypes, in sarcomas remain unknown. In this study, we found that TUBB3 overexpression induced higher tumor growth in both in vitro and clinical samples. Because high tumor growth is one of the major causes to make tumor aggressiveness, we considered that the patients with leiomyosarcoma which expressed high TUBB3 resulted in poorer prognosis than did the patients with low TUBB3. Tumor cell growth is also a major factor for drug resistance. In this study, the eribulin resistant cell line which expresses higher TUBB3 tends to grow more rapidly than does the parent cell line and the knockdown of TUBB3 by siRNA resulted in a trend toward the inhibition of cell growth. So eribulin resistance by TUBB3 overexpression may be partially due to the increase of cell proliferation. Our study is the first report to show expression levels in a large group of leiomyosarcoma patients and to elucidate the relationship with TUBB3 expression level and prognosis of the patients with leiomyosarcoma. Importantly, the level of TUBB3 expression was heterogeneous in leiomyosarcoma patients and it is reasonable to infer that sensitivity to eribulin might be negatively associated with TUBB3 expression level.

In this study, we clearly reveal the critical role of TUBB3 in the acquisition of eribulin resistance in a leiomyosarcoma cell line. Because the level of TUBB3 expression was variable in leiomyosarcoma patients, TUBB3 has the potential to be a biomarker for selection of drug treatment. Namely, if overexpression of TUBB3 is observed, this suggests that eribulin would be ineffective and alternative drugs should be considered. Elucidation of the mechanism of TUBB3 overexpression may lead to new therapeutic strategies to overcome eribulin resistance in the future.

## 5. Conclusion

We developed an eribulin-resistant leiomyosarcoma cell line to investigate the mechanism underlying resistance and found that TUBB3 overexpression induced chemoresistance to eribulin and knockdown of TUBB3 expression by siRNA led to chemosensitization to eribulin in the resistant cell line. This is the first report to reveal the critical role of TUBB3 in the acquisition of eribulin resistance in a leiomyosarcoma. We also found that the level of TUBB3 expression was variable in leiomyosarcoma patients. TUBB3 has the potential to be a therapeutic target to overcome eribulin resistance and a biomarker for selection of drug treatment.

## Data Availability

The data used to support the findings of this study included information from clinical samples and so cannot be made freely available because of patient privacy.

## Supplementary Materials

Supplementary Materials Supplementary Figure 1: the immunohistochemical staining of Pgp in two patients with leiomyosarcoma. The immunohistochemical staining of PGP was performed for a sample of two patients used in Figure 4(a). The left panel represents the tissue samples from the patient who continued to have stable disease (SD) 12 weeks after initial treatment with eribulin. The right panel represents the tissue samples from the patient who resulted in progressive disease (PD) after treatment with eribulin. We only have a slight Pgp expression in both samples. Scale bar represents 20 *μ*m in (a) and (b).
