# Supplementary figures and images for: Class III β-Tubulin Overexpression Induces Chemoresistance to Eribulin in a Leiomyosarcoma Cell Line
Source: Anal Cell Pathol (Amst). 2018 Jun 21;2018:8987568. doi: 10.1155/2018/8987568 (PMC6033248; doi:10.1155/2018/8987568)

Supplementary Fig. 1

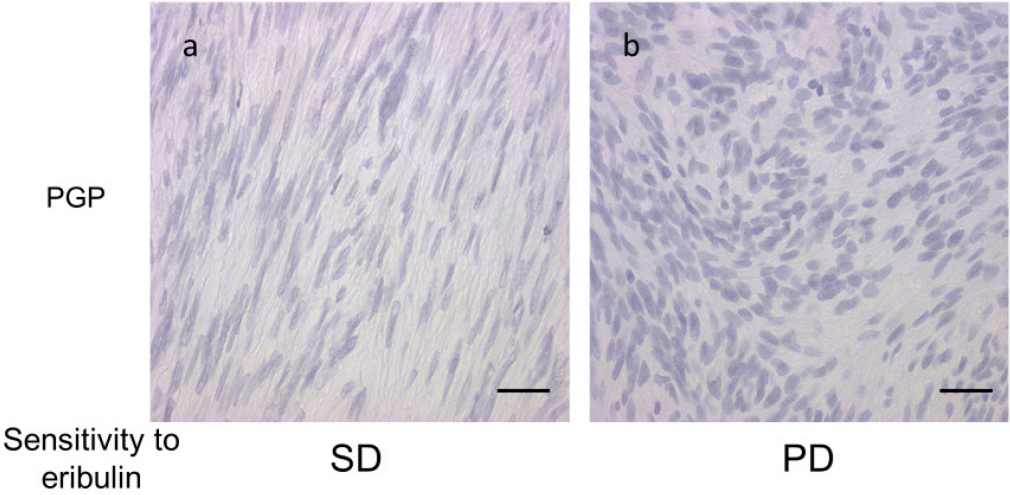

Supplement: Supplementary Materials — Supplementary Figure 1: the immunohistochemical staining of Pgp in two patients with leiomyosarcoma. The immunohistochemical staining of PGP was performed for a sample of two patients used in Figure 4(a). The left panel represents the tissue samples from the patient who continued to have stable disease (SD) 12 weeks after initial treatment with eribulin. The right panel represents the tissue samples from the patient who resulted in progressive disease (PD) after treatment with eribulin. We only have a slight Pgp expression in both samples. Scale bar represents 20 μm in (a) and (b). [file 8987568.f1.pdf]
